# Valproic Acid Enhances Venetoclax Efficacy in Targeting Acute Myeloid Leukemia

**DOI:** 10.3390/diseases13010010

**Published:** 2025-01-08

**Authors:** Renshi Kawakatsu, Kenjiro Tadagaki, Kenta Yamasaki, Yasumichi Kuwahara, Tatsushi Yoshida

**Affiliations:** 1 Department of Biochemistry and Molecular Biology, Graduate School of Medical Science, Kyoto Prefectural University of Medicine, Kyoto 602-8566, Japan; 2 Department of Kyoto Pediatric Community-Based Medicine, Graduate School of Medical Science, Kyoto Prefectural University of Medicine, Kyoto 602-8566, Japan

**Keywords:** acute myeloid leukemia, valproic acid, apoptosis, Bcl-2, Bax, Bak

## Abstract

Background: Acute myeloid leukemia (AML) is a common and aggressive form of leukemia, yet current treatment strategies remain insufficient. Venetoclax, a BH3-mimetic approved for AML treatment, induces Bcl-2-dependent apoptosis, though its therapeutic efficacy is still limited. Therefore, new strategies to enhance the effect of venetoclax are highly sought. Valproic acid (VPA), commonly used for epilepsy, has also been studied for potential applications in AML treatment. Methods: AML cells were treated with venetoclax, with or without VPA. Cell viability was assessed using the trypan blue dye exclusion assay, while cell cycle progression was analyzed by flow cytometry. The expression of pro-apoptotic proteins Bax and Bak was measured by RT-qPCR. Results: Venetoclax and VPA individually had only mild effects on AML cell proliferation. However, their combination significantly inhibited cell growth and triggered pronounced cell death. This combination also led to the cleavage of poly (ADP-ribose) polymerase (PARP), a substrate of caspases, indicating activation of apoptosis. VPA treatment upregulated the expression of Bax and Bak, further supporting apoptosis induction. The cell death induced by the venetoclax–VPA combination was predominantly apoptotic, as confirmed by the near-complete blockade of cell death by a pan-caspase inhibitor. Conclusions: Our study demonstrates that VPA enhances venetoclax-induced apoptosis in AML cell lines, providing a novel role for VPA and suggesting a promising combinatory strategy for AML treatment. These findings offer valuable insights into potential clinical applications of venetoclax and VPA in AML management.

## 1. Introduction

One of the primary challenges in public health is the effective treatment of malignant tumors. Acute myeloid leukemia (AML) is the most prevalent form of adult leukemia [1], with a 5-year overall survival rate still below 50% [2,3]. A significant number of AML patients experience relapse and develop resistance to chemotherapy [4,5,6], highlighting the need for novel therapeutic strategies.

Inducing cell death is a promising approach for tumor suppression, and numerous anti-tumor agents targeting cell death pathways have been developed [7,8,9]. Cell death is categorized into three main types based on its form and molecular mechanisms: Type I (apoptosis), Type II (autophagic cell death), and Type III (necrotic cell death) [10,11]. Recent research has identified additional types of cell death, such as necroptosis [9,12], ferroptosis [13], and pyroptosis [14]. Apoptosis is primarily executed via the mitochondria, which plays a key role in this process [15,16,17]. Apoptotic signals increase mitochondrial outer membrane permeability (MOMP), a process regulated by the B-cell leukemia gene-2 (Bcl-2) family proteins. Pro-apoptotic factors, such as Bcl-2-associated X protein (Bax) and Bcl-2 homologous antagonist/killer (Bak), are thought to oligomerize in response to apoptotic signals, accumulating in mitochondria to form pores that increase MOMP [18]. Bcl-2 and Bcl-XL, on the other hand, bind to the BH3 domain of Bax or Bak to inhibit their oligomerization, functioning as anti-apoptotic factors. Once MOMP is elevated, cytochrome C is released from the mitochondria into the cytoplasm, subsequently activating cysteine aspartate-specific proteases (caspases) that lead to apoptosis.

Navitoclax (ABT-263), a small molecule that mimics the BH3 domain, was developed as an anti-tumor agent [19,20]; it binds to Bcl-2, Bcl-XL, and Bcl-w to induce pro-apoptotic signaling. Although navitoclax demonstrated efficacy in clinical trials for chronic lymphocytic leukemia (CLL) and small-cell lung cancer, it caused dose-limiting thrombocytopenia due to Bcl-XL’s role in platelet production [21,22]. Consequently, venetoclax (ABT-199), a selective Bcl-2 inhibitor with reduced thrombocytopenic effects, was developed [23]. In a phase III clinical trial, venetoclax in combination with rituximab extended progression-free survival to 57.3% compared to 4.6% with rituximab alone and increased overall survival to 85.3% versus 66.8% [24]. For AML, phase III clinical trials of venetoclax combined with azacitidine or low-dose cytarabine (LDAC) demonstrated improved median overall survival [azacitidine + venetoclax (14.7 months) vs. azacitidine alone (9.6 months); venetoclax + LDAC (7.2 months) vs. LDAC alone (4.1 months)] [25,26]. In 2016, the U.S. Food and Drug Administration (FDA) approved venetoclax for patients with relapsed or refractory CLL. In 2018, venetoclax received approval for patients aged 75 and older with newly diagnosed AML or those ineligible for intensive induction chemotherapy due to comorbidities. However, the efficacy of venetoclax combined with azacitidine or cytarabine remains limited, prompting exploration of new combination therapies [27,28,29]. Thus, developing strategies to enhance the efficacy of venetoclax is necessary.

Valproic acid (VPA), a short-chain fatty acid with histone deacetylase inhibitory (HDACi) activity, has been used clinically for many years, primarily in the treatment of epilepsy [30,31,32,33]. VPA is also approved for managing bipolar disorder and preventing migraines and is listed on the WHO Model List of Essential Medicines. It induces differentiation and apoptosis in AML cells, either alone or in combination with agents such as all-trans retinoic acid [34,35,36]. Phase II and III clinical studies using VPA in combination with other agents for AML treatment have shown beneficial outcomes [37,38,39,40,41,42,43,44,45].

Therefore, we hypothesized that VPA, in combination with venetoclax, would exert synergistic effects in AML treatment.

This study demonstrates that VPA enhances the efficacy of venetoclax in AML cells by promoting caspase-dependent apoptotic activity.

## 2. Materials and Methods

### 2.1. Cell Culture

The acute myeloid leukemia (AML) cell line KG-1 and HL-60 was obtained from Riken BioResource Center and cultured in RPMI-1640 (FUJIFILM Wako Pure Chemical, Osaka, Japan) supplemented with 10% fetal bovine serum (FBS) (Sigma-Aldrich; Merck KGaA, Burlington, MA, USA). The SKNO-1 AML cell line, obtained from the JCRB cell bank, was cultured in RPMI-1640 with 10% FBS and 10 ng/ml recombinant human granulocyte-macrophage colony-stimulating factor (GM-CSF) (Pepro Tech, Rocky Hill, NJ, USA). The chronic myeloid leukemia cell line K562 was acquired from Riken BioResource Center and cultured in RPMI-1640 with 10% FBS. All cell lines were maintained at 37 °C in a humidified atmosphere with 5% CO_2_.

### 2.2. Reagents

Venetoclax (also known as ABT-199) was purchased from Selleckchem (Houston, TX, USA). The pan-caspase inhibitor Q-VD-OPh was obtained from MedChemExpress (Monmouth Junction, NJ, USA) and applied to cells at a concentration of 20 µM. Sodium valproate, used as VPA in this study, was purchased from FUJIFILM Wako Pure Chemical. All reagents were dissolved in dimethyl sulfoxide (DMSO) as a solvent.

### 2.3. Western Blotting

Cells were lysed in RIPA buffer with aprotinin, and the supernatant was collected as the cell lysate following centrifugation to remove the pellet. Proteins from the cell lysate were separated by 7.5% sodium dodecyl sulfate (SDS)-polyacrylamide gel electrophoresis (PAGE) and transferred to a nitrocellulose membrane. The membrane was probed with poly (ADP-ribose) polymerase (PARP) antibody (#9542, Cell Signaling Technology, Danvers, MA, USA) or β-actin antibody (PM053, Medical and Biological Laboratories, Tokyo, Japan). Signals were visualized using ECL Western blotting detection reagents (GE Healthcare, Chicago, IL, USA) and an ImageQuant LAS500 (GE Healthcare, Chicago, IL, USA). Quantification of cleaved PARP was performed by Image J software Version 1.54m (https://imagej.net/ij/).

### 2.4. RT-qPCR

Total RNA was extracted from cells using Isogen II reagent (Nippongene, Tokyo, Japan). Equal amounts of RNA were reverse-transcribed to cDNA with Superscript IV reverse transcriptase (Thermo Fisher Scientific, Waltham, MA, USA). The cDNA was amplified using Thunderbird SYBR qPCR mix (Toyobo Life Science, Osaka, Japan) on a StepOne Plus real-time PCR system (Thermo Fisher Scientific) with the following primers:

**Human BAX:** Forward 5′-TCAGGATGCGTCCACCAAGAAG, Reverse 5′-TGTGTCCACGGCGGCAATCATC

**Human BAK1**: Forward 5′-ATGGTCACCTTACCTCTGCAA, Reverse 5′-TCATAGCGTCGGTTGATGTCG

**Human Bcl-2**: Forward 5′-TGGGATGCCTTTGTGGAACTGTA, Reverse 5′-ATATTTGTTTGGGGCAGGCATGT

**Human Actin:** Forward 5′-GCTGTGCTACGTCGCCCTG, Reverse 5′-GGAGGAGCTGGAAGCAGCC

These primers were synthesized by Eurofins Genomics (Tokyo, Japan).

### 2.5. Cell Counts

Cells were mixed with an equal volume of trypan blue solution and examined under an Olympus CK40 inverted microscope (Olympus, Tokyo, Japan) using a Bürker-Türk counting chamber (Erma, Saitama, Japan). Cells stained blue were counted as dead, as they could not exclude the dye from their cytosol.

### 2.6. Flow Cytometry Analysis

Cells were harvested by centrifugation, and the cell pellet was resuspended in propidium iodide (PI) solution (PBS, 0.1% Triton X-100, 10 µg/ml PI). DNA content analysis was performed using BD FACS Canto II and BD FACS Diva software v6.1.3 (BD Biosciences, Franklin Lakes, NJ, USA).

### 2.7. Statistical Analysis

Data was analyzed using one-way ANOVA followed by Tukey’s post hoc test with GraphPad Prism 10 (Dotmatics, Boston, MA, USA).

## 3. Results

### 3.1. VPA Enhances the Effect of Venetoclax on Inhibition of AML Cell Growth

**Figure 1:**
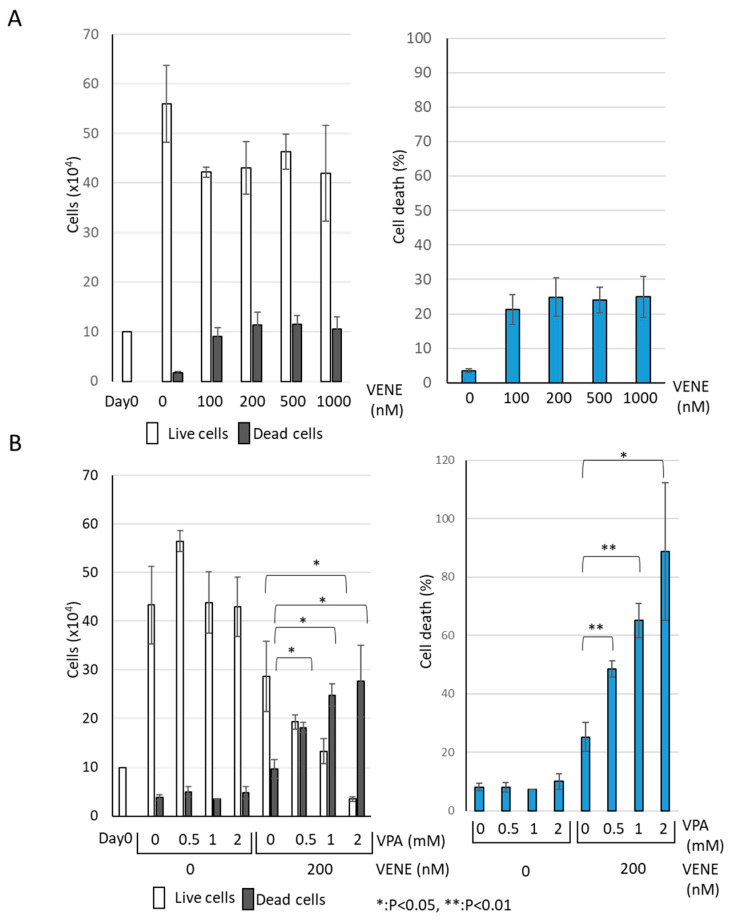
**VPA Enhances Venetoclax’s Effect on AML Cell Growth Inhibition.** Trypan blue dye exclusion assay was performed on the AML cell line KG-1, treated with venetoclax alone at indicated concentrations (**A**) and with a combination of venetoclax and VPA (**B**) for 48 h. Left panels: cell counts of live cells and dead cells; right panels: the percentage of cell death for total cells. Data represent mean ± S.D. (n = 3).

We examined the effect of venetoclax on the growth of AML cell line KG-1 using a trypan blue dye exclusion assay. Venetoclax alone resulted in a slight decrease in the number of live cells (Figure 1A). Although venetoclax treatment increased the number of trypan-blue-stained dead cells, the effect was minimal, with only about 10% cell death observed even at 1000 nM. When used individually, neither VPA nor venetoclax caused any drastic change in the number of live or dead cells (Figure 1B). However, when VPA was combined with venetoclax, a marked increase in cell death was observed. The combination of VPA at 2 mM and venetoclax at 200 nM significantly induced cell death in the majority of cells. These findings indicate that venetoclax alone has a limited effect on KG-1 cell viability, while VPA substantially enhances the growth-inhibitory effect of venetoclax.

### 3.2. Combined Treatment of Venetoclax and VPA Alters Cell Morphology

**Figure 2:**
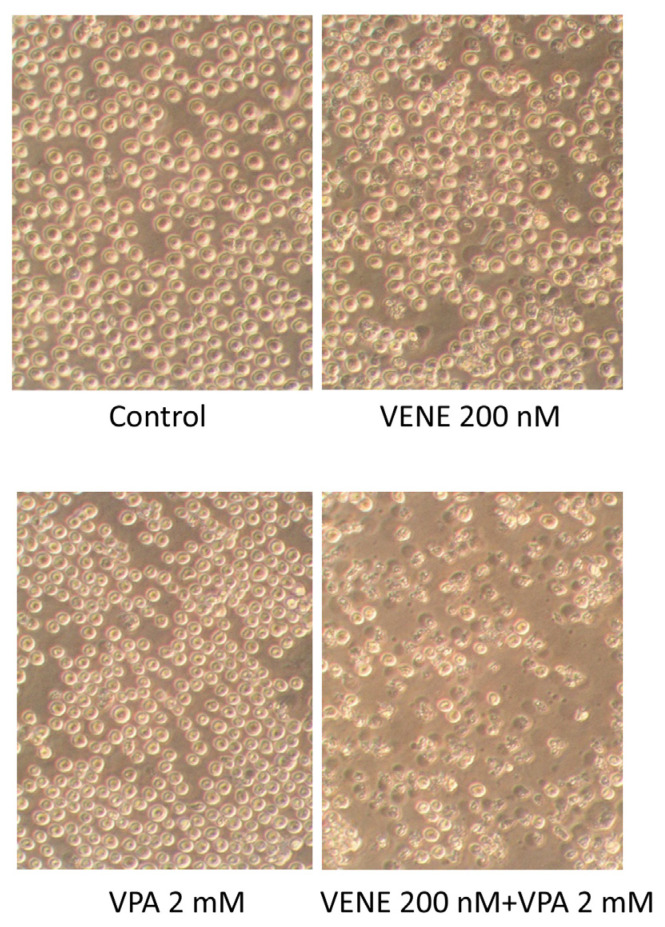
**Venetoclax treatment combined with VPA alters cell morphology.** KG-1 cells were treated with venetoclax with or without VPA for 48 h, and cell morphologies were observed under a phase-contrast microscope.

Cell morphology was observed under a microscope following treatment of KG-1 cells with venetoclax, with or without VPA (Figure 2). Neither VPA nor venetoclax alone caused any significant morphological changes. However, the addition of VPA to venetoclax treatment resulted in numerous cells exhibiting shrunken and broken morphologies. These morphological changes are consistent with the cell growth inhibition observed in Figure 1.

### 3.3. Venetoclax and VPA Induce Sub-G1 Population and Reduce Other Cell Cycle Phases

**Figure 3:**
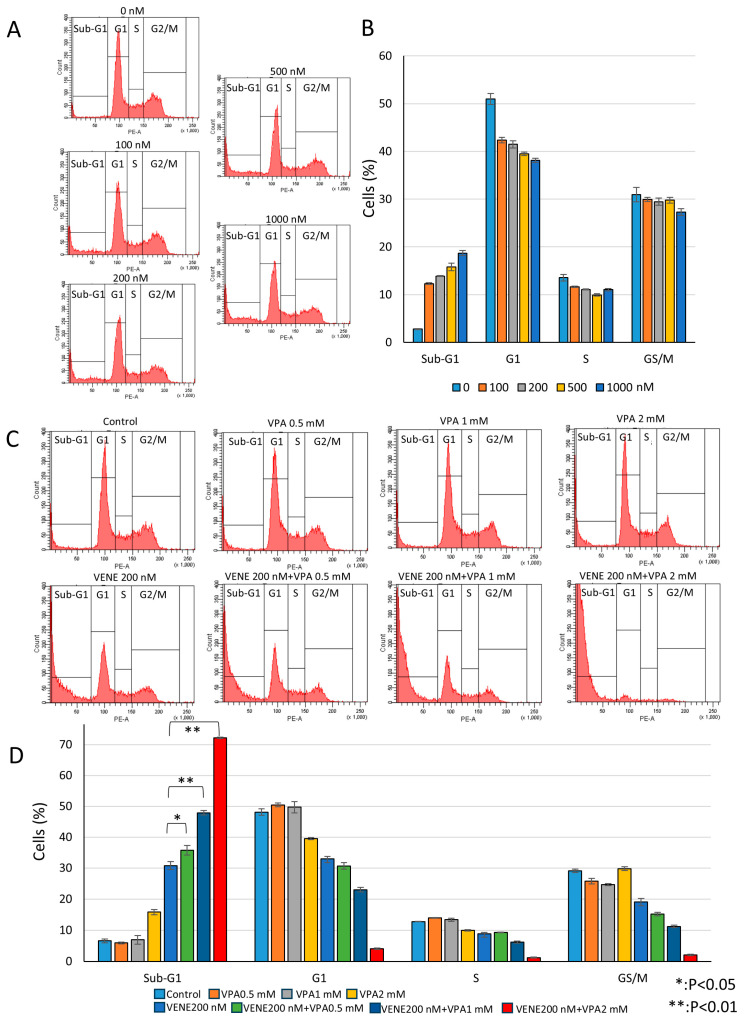
**Venetoclax and VPA induce sub-G1 population and decrease other cell cycle phases.** Cell cycle analysis of PI-stained KG-1 cells treated with venetoclax alone (**A**) and in combination with VPA for 48 h (**C**). Representative histograms (**A**,**C**) and a bar graph (**B**,**D**) are shown. Data represent mean ± S.D. (n = 3).

Next, we performed cell cycle analysis using flow cytometry. Venetoclax alone slightly reduced the G1, S, and G2/M populations in a dose-dependent manner while weakly increasing the sub-G1 population (Figure 3A,B). The sub-G1 population, representing cells with degraded DNA, indicates cell death. When combined with VPA, venetoclax significantly induced the sub-G1 population (Figure 3C,D), with approximately 50% and 70% of cells killed at VPA concentrations of 1 mM and 2 mM, respectively. These results suggest that VPA acts as an enhancer of venetoclax efficacy by promoting sub-G1 population induction. Further analysis was conducted on other AML cell lines, SKNO-1 carrying t(8;21) (Supplementary Figure S1) and HL-60 belonging to acute promyelocytic leukemia (Supplementary Figure S2). Similar to KG-1 cells, SKNO-1 and HL-60 cells were almost unaffected by venetoclax or VPA alone, but the combination treatment induced substantial cell death, as indicated by an increased sub-G1 population and decreased G1, S, and G2/M populations. In addition, another HDAC inhibitor, trichostatin A (TSA), also enhanced venetoclax efficacy on cell death induction in KG-1 cells (Supplementary Figure S3).

To investigate whether the combined effect of venetoclax and VPA was specific to AML cells, we examined K562 cells, a chronic myeloid leukemia (CML) cell line expressing the BCR-ABL fusion protein due to t(9;22) chromosome translocation. While cell growth was partially reduced, the combined venetoclax and VPA treatment did not increase cell death in K562 cells (Supplementary Figure S4A). Furthermore, the combination treatment, which induced the sub-G1 population in AML cell lines, did not cause the sub-G1 population in K562 cells (Supplementary Figure S4B,C). These results suggest that the combined treatment of venetoclax and VPA specifically induces cell death in AML cells but not in CML K562 cells.

### 3.4. Caspase Inhibitor Significantly Blocks Cell Death Induced by Venetoclax and VPA Combination

**Figure 4:**
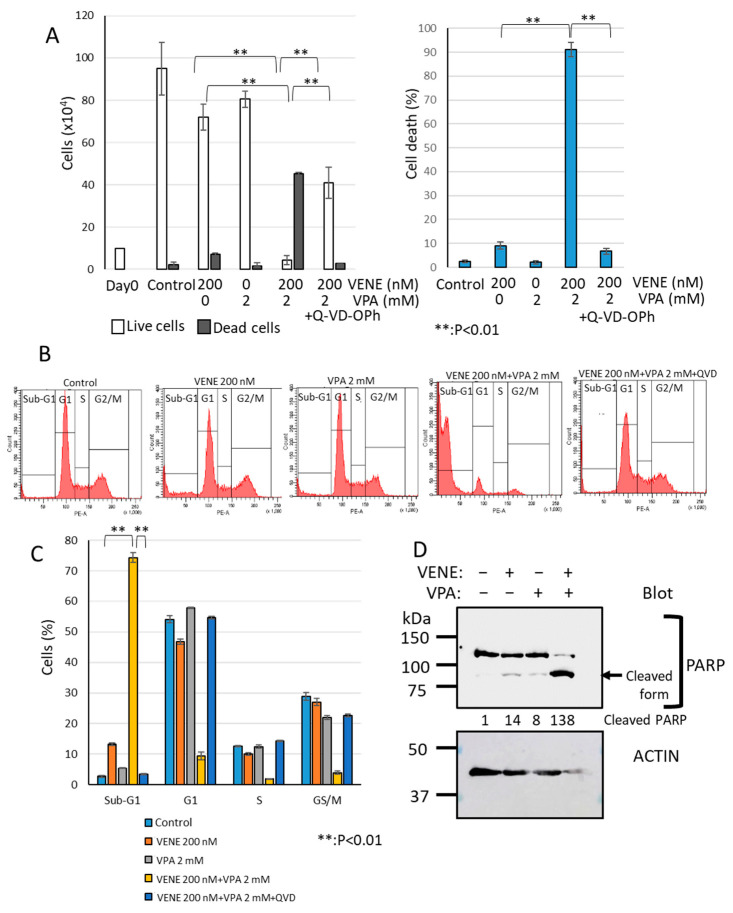
**Caspase inhibitor Q-VD-OPh blocks cell death induced by venetoclax and VPA.** KG-1 cells were treated with venetoclax and VPA with or without Q-VD-OPh for 48 h. (**A**): Trypan blue dye exclusion assay; left panel, cell counts of live cells and dead cells; right panel, the percentage of cell death for total cells. (**B**,**C**): Cell cycle analysis of PI-stained cells by flow cytometry. Representative histograms (**B**) and a bar graph (**C**) are shown. Data represent mean ± S.D. (n = 3). Western blot analysis was performed for PARP and actin (**D**). Cleaved PARP generated by caspase activation is indicated. Quantification of cleaved PARP is added to the blot.

We next investigated the cell death type induced by the combined treatment of venetoclax and VPA using the broad-spectrum caspase inhibitor Q-VD-OPh, known to block apoptosis effectively [46,47,48]. As shown in Figure 4A, venetoclax or VPA alone only slightly induced cell death, whereas the combination treatment drastically increased dead cells. When Q-VD-OPh was added, cell death induced by venetoclax and VPA was almost completely inhibited, reducing dead cells to control levels. Flow cytometry analysis further confirmed that Q-VD-OPh eliminated the large sub-G1 population induced by venetoclax and VPA combination (Figure 4B,C). Western blot analysis demonstrated that PARP, a caspase substrate, was cleaved by the venetoclax and VPA combination (Figure 4D), indicating apoptosis induction. These findings suggest that venetoclax and VPA primarily induce apoptosis-dependent cell death.

### 3.5. VPA Induces Bax and Bak Expression

**Figure 5:**
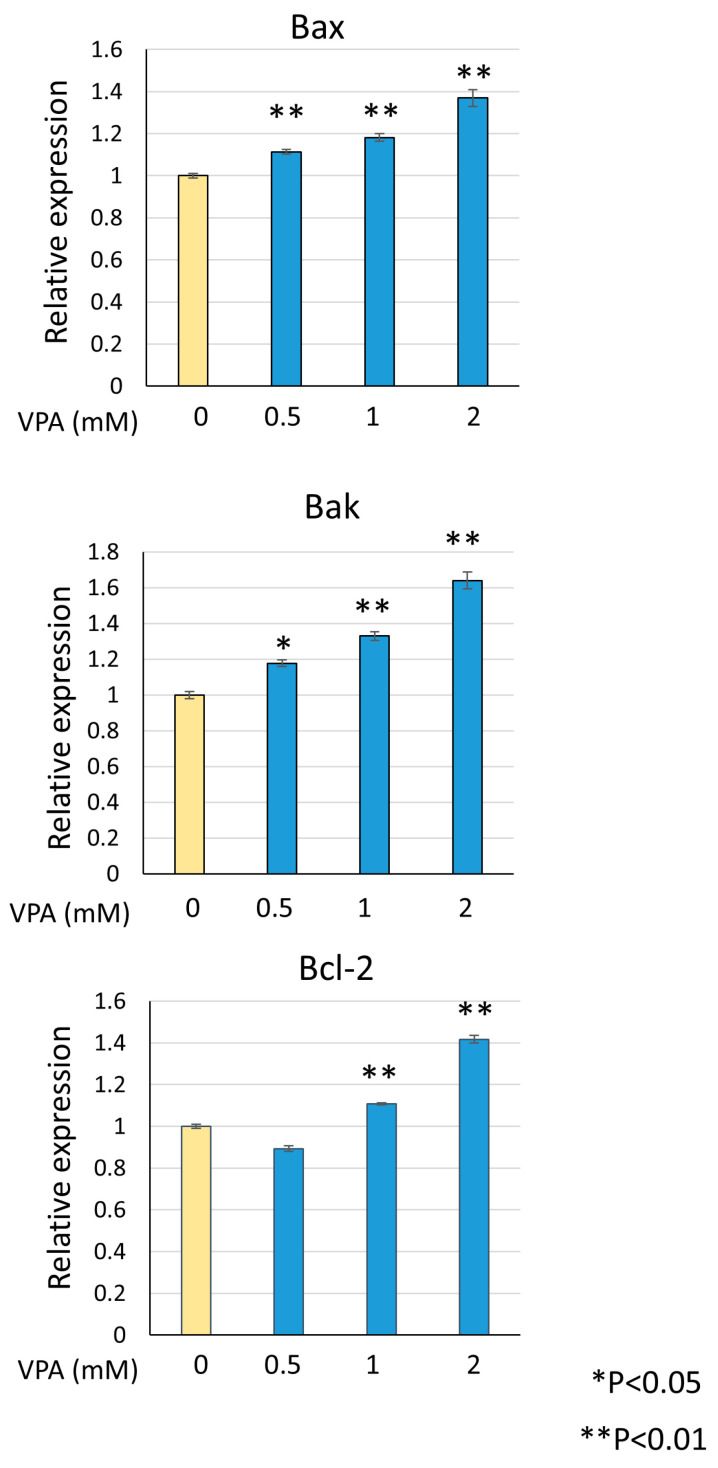
**VPA induces Bax and Bak expression.** KG-1 cells were treated with indicated VPA concentrations, total RNA was extracted, and RT-qPCR was performed for Bax, Bak, and Bcl-2. Data were normalized to actin expression and represent mean ± S.D. (n = 3).

Venetoclax directly binds to Bcl-2 to induce apoptosis, but upstream apoptotic stimuli are required to enhance its activity. During apoptosis, Bax or Bak triggers mitochondrial outer membrane permeabilization (MOMP) and releases cytochrome C into the cytosol, leading to caspase activation and apoptosis. Bcl-2 inhibits apoptosis by binding to Bax and Bak at the mitochondria. To investigate the mechanism by which venetoclax and VPA combination induce cell death, we examined the effect of VPA on Bax and Bak expression levels. As shown in Figure 5, VPA dose-dependently upregulated Bax and Bak mRNA, with a concomitant increase in Bcl-2 expression. The upregulation of Bax and Bak provides a mechanism by which VPA enhances venetoclax-induced apoptosis.

## 4. Discussion

In this study, we demonstrated that valproic acid (VPA) enhances venetoclax-induced cell death, whereas venetoclax alone induces only limited cell death in AML cells. Using the pan-caspase inhibitor Q-VD-OPh, we confirmed that the cell death induced by the VPA–venetoclax combination is primarily apoptotic. Other methods, such as the detection of annexin V on the cell surface, are also good to confirm apoptosis induction. Although venetoclax has been approved for AML treatment, its effect remains limited. Our findings suggest that combining venetoclax with VPA could improve its efficacy. Previous studies have explored various agents as venetoclax combination partners [27,28,29], with VPA standing out due to its clinical safety profile from long-term use in epilepsy treatment. VPA is also orally administrable and has been tested in clinical trials for AML in combination with other agents like ATRA. Thus, combining venetoclax with VPA represents a promising strategy for AML treatment.

AML has shown adaptive resistance to venetoclax combined with hypomethylating agents or low-dose cytarabine (LDAC) [49,50]; however, pairing venetoclax with VPA may potentially overcome this resistance. Venetoclax is also under investigation in a phase III trial for multiple myeloma [51] and phase II trials for B-cell lymphoma and breast cancer. The VPA–venetoclax combination could, therefore, represent a viable treatment option for other malignancies as well as AML.

**Figure 6:**
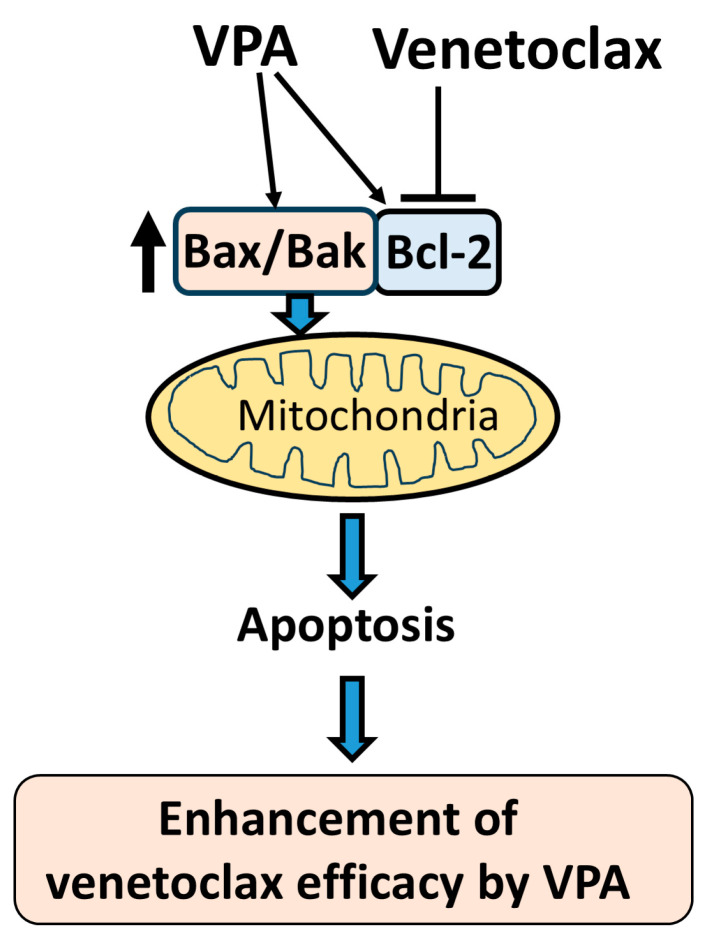
**Schematic diagram of proposed mechanism.** This schematic illustrates the proposed mechanism by which VPA enhances the efficacy of venetoclax in inducing apoptosis. VPA upregulates the expression of Bax, Bak, and Bcl-2. Venetoclax inhibits Bcl-2, which releases the increased Bax and Bak from Bcl-2 binding. The free Bax and Bak then target the mitochondria, triggering mitochondrial outer membrane permeabilization (MOMP) and inducing apoptosis. This cooperative effect of VPA and venetoclax results in an enhanced apoptotic response in AML cells.

Regarding the molecular mechanism by which VPA enhances venetoclax efficacy, we showed that VPA upregulates Bax and Bak expression. As venetoclax is a Bcl-2 inhibitor, it requires an apoptotic trigger to activate its function effectively, suggesting that agents like VPA, which induce Bax and Bak, are suitable partners in AML treatment (Figure 6). Our data showed that VPA alone induced only a slight apoptotic effect, possibly due to simultaneous Bcl-2 upregulation along with Bax and Bak. However, even with increased Bcl-2 levels, the venetoclax-mediated Bcl-2 blockade allows VPA-driven Bax and Bak upregulation to facilitate apoptotic signaling. Since KG-1, SKNO-1, and HL-60 cells were effectively killed by the combination of venetoclax and VPA, these AML cell lines are unlikely to have mutations that render them insensitive to venetoclax. It will be important to examine whether these cells have mutations in Bax and/or Bak that are associated with the induction of apoptosis. We demonstrate up-regulation of Bax and Bak transcripts, but detection of Bax and Bak proteins is also required. Although we demonstrate caspase activation by detecting PARP cleavage, it is also necessary to confirm the activation of the caspases themselves.

Tumor necrosis factor-related apoptosis-inducing ligand (TRAIL) is an anti-tumor cytokine, and recombinant TRAIL protein shows promise as an anti-tumor agent [52,53,54]. Previously, we reported that VPA sensitizes AML cells to TRAIL-induced apoptosis, though the mechanism remained unclear [55]. Our current study suggests that VPA’s ability to upregulate Bax and Bak may explain this sensitization to TRAIL. Similarly, we recently reported that sodium butyrate, another HDAC inhibitor, upregulates Bax and Bak and enhances venetoclax efficacy [28]. Therefore, VPA’s HDAC inhibitory function may contribute to its synergy with venetoclax. Blocking of Bax and Bak would be required to more clearly show the relationship in apoptosis of the synergistic effects. In addition, other members of the Bcl-2 family and inhibitor of apoptosis (IAP) proteins regulate apoptosis; thus, the expression of more factors will need to be confirmed to comprehensively investigate the mechanism of apoptosis induction.

## 5. Conclusions

This study demonstrates that VPA strengthens venetoclax-induced apoptosis, highlighting a promising strategy for AML treatment.

## Data Availability

The datasets generated during this study are available from the corresponding author upon reasonable request.

## Supplementary Materials

The following supporting information can be downloaded at: https://www.mdpi.com/article/10.3390/diseases13010010/s1, Figure S1: Combination of venetoclax with VPA drastically induces cell death in SKNO-1 cells. Figure S2: Combination of venetoclax with VPA drastically induces cell death in HL-60 cells. Figure S3: Combination of venetoclax with TSA induces cell death in KG-1 cells. Figure S4: Combination of venetoclax and VPA does not induce cell death in K562 cells.
