# Supplementary material for: Valproic Acid Enhances Venetoclax Efficacy in Targeting Acute Myeloid Leukemia
Source: Diseases. 2025 Jan 8;13(1):10. doi: 10.3390/diseases13010010 (PMC11764158; doi:10.3390/diseases13010010)
Supplement: Supplementary file 1 [file diseases-13-00010-s001.zip › diseases-3336273-supplementary.pdf]

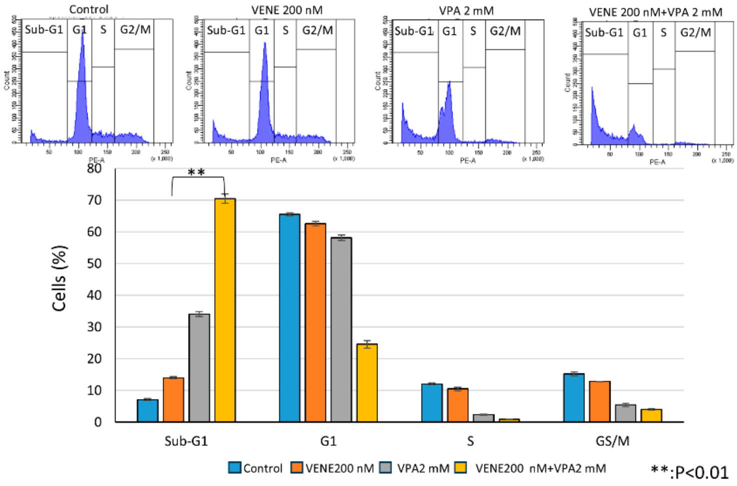

Figure S1. Combination of Venetoclax with VPA Drastically Induces Cell Death in SKNO-1 Cells. SKNO-1 cells were treated with the indicated concentrations of venetoclax, with or without VPA, for 48 hours. Cell cycle analysis was performed on PI-stained cells using a flow cytometer. Representative histograms and a bar graph showing triplicate data are presented. Data are shown as mean ± S.D. (n=3).

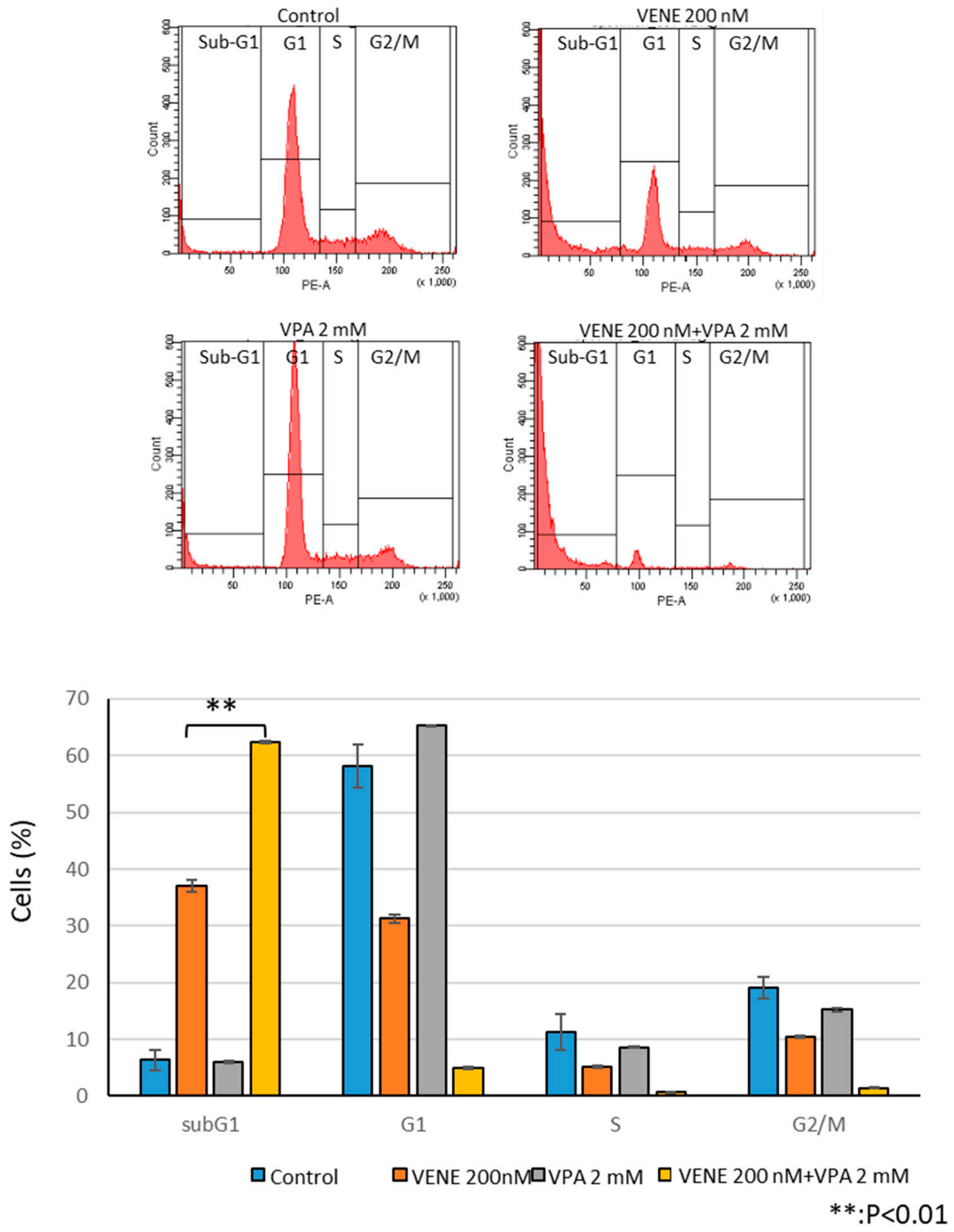

Figure S2. Combination of Venetoclax with VPA Drastically Induces Cell Death in HL-60 Cells. HL-60 cells were treated with venetoclax, with or without VPA, for 48 hours. Cell cycle was analyzed by PI-staining and flow cytometry. Representative histograms and a bar graph showing triplicate data are presented. Data are shown as mean ± S.D. (n=3).

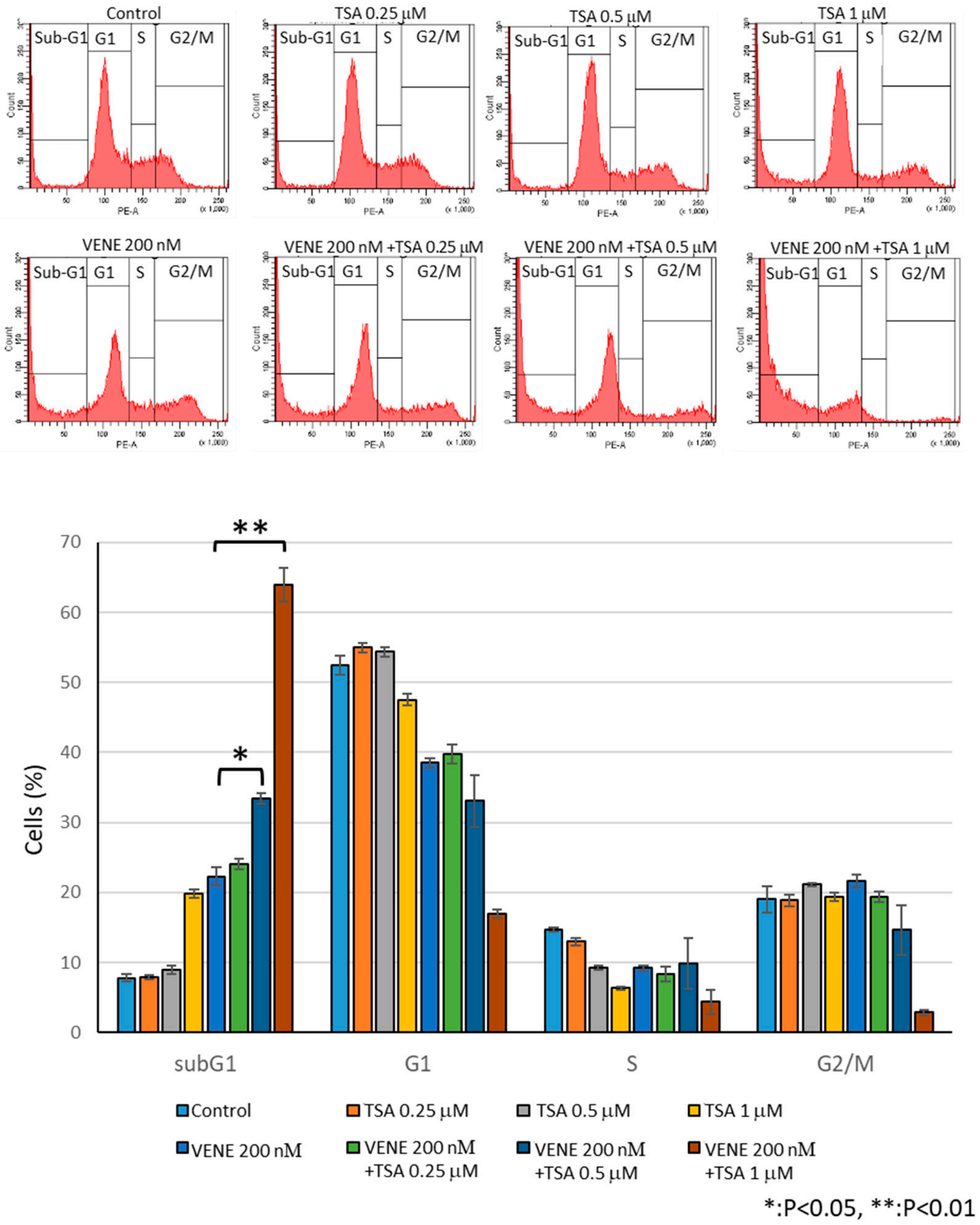

Figure S3. Combination of Venetoclax with TSA Induces Cell Death in KG-1 Cells. KG-1 cells were treated with venetoclax, with or without TSA, for 48 hours. Cell cycle was analyzed using flow cytometry. Representative histograms and a bar graph showing triplicate data are presented. Data are shown as mean ± S.D. (n=3).

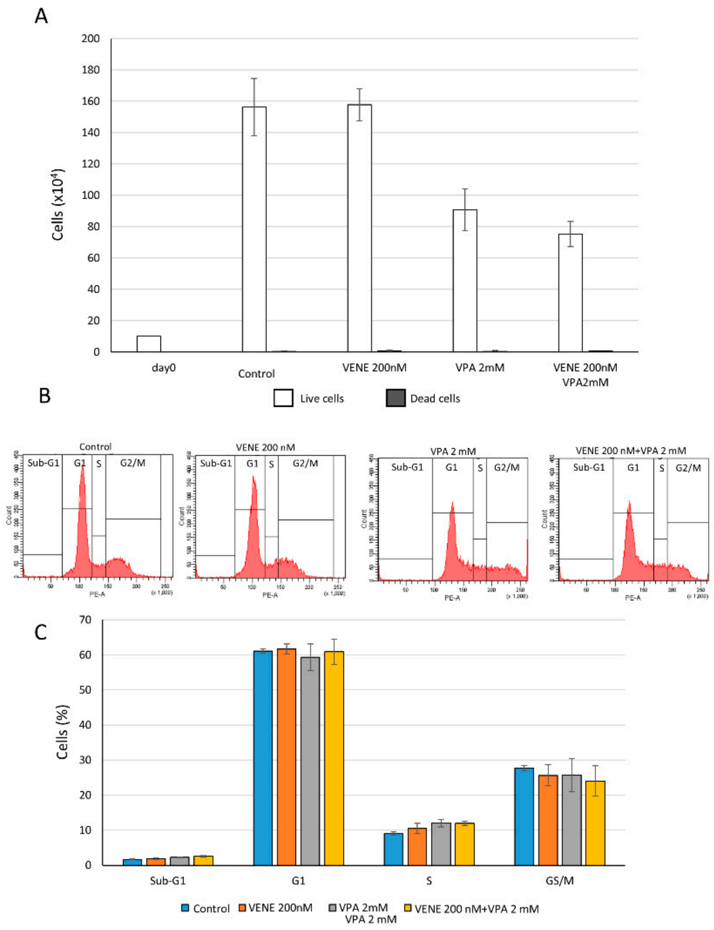

Figure S4. Combination of Venetoclax and VPA Does Not Induce Cell Death in K562 Cells. The chronic myeloid leukemia cell line K562 was treated with a combination of venetoclax and VPA for 48 hours. A: Trypan blue dye exclusion assay was used to assess cell viability. B and C: Cell cycle analysis was performed on PI-stained cells using a flow cytometer. Representative histograms (B) and a bar graph (C) are presented. Data are shown as mean ± S.D. (n=3).
